# Effects of Blindfold on Leadership in Pediatric Resuscitation Simulation: A Randomized Trial

**DOI:** 10.3389/fped.2019.00010

**Published:** 2019-02-14

**Authors:** Michael Buyck, Sergio Manzano, Kevin Haddad, Anne-Catherine Moncousin, Annick Galetto-Lacour, Katherine Blondon, Oliver Karam

**Affiliations:** ^1^Department of Pediatric Emergency, Children's Hospital of Geneva, Geneva, Switzerland; ^2^SimKids, Children's Hospital of Geneva, Geneva, Switzerland; ^3^Interprofessional Simulation Center, University of Geneva, Geneva, Switzerland; ^4^Medical Directorate, University Hospitals of Geneva, Geneva, Switzerland; ^5^Pediatric Critical Care Unit, Children's Hospital of Geneva, Geneva, Switzerland; ^6^Pediatric Critical Care Unit, Children's Hospital of Richmond, Richmond, VA, United States

**Keywords:** pediatric, emergency medicine, simulation-based training, non-clinical skills, leadership

## Abstract

**Background:** Pediatric resuscitations are rare events. Simulation-based training improves clinical and non-clinical skills, as well as survival rate. We assessed the effectiveness of using blindfolds to further improve leadership skills in pediatric simulation-based training.

**Methods:** Twelve teams, each composed of 1 pediatric emergency fellow, 1 pediatric resident, and 2 pediatric emergency nurses, were randomly assigned to the blindfold group (BG) or to the control group (CG). All groups participated in one session of five simulation-based resuscitation scenarios. The intervention was using a blindfold for the BG leader for the scenarios B, C, and D. Three evaluators, who were blinded to the allocation, assessed leadership skills on the first and last video-recorded scenarios (A and E). Questionnaires assessed self-reported changes in stress and satisfaction about skills after the first and the last scenarios.

**Results:** Improvement in leadership skills doubled in the BG compared with the CG (11.4 vs. 5.4%, *p* = 0.04), whereas there was no increase in stress or decrease in satisfaction.

**Conclusion:** Blindfold could be an efficient method for leadership training during pediatric resuscitation simulated scenarios. Future studies should further assess its effect at a follow-up and on clinical outcomes after pediatric resuscitation.

## What is already known on this subject

- As pediatric resuscitations are rare events, simulation-based training have been developed to train the teams who respond to these situations.
- Most teaching techniques focus on improving clinical skills.
- There is very little evidence on how to improve non-clinical skills, such as leadership skills.

## What This Study Adds

- Our study suggests that blindfolding the leader during simulation sessions might improve leadership skills training.
- Our study has identified an innovative way of improving leadership skills.

## Introduction

Pediatric cardiac arrests are rare events in the out-of-hospital (8 events per 100,000 person year) and in-hospital pediatric population (20 events per 100,000 person year) (1, 2). These low incidences do not provide sufficient exposure to allow teams to master these situations. Simon and Sullivan suggested a correlation between emergency physicians' degree of comfort with potentially life-saving skills and frequency of critical situations (3). Similarly, 50% of residents felt inadequately trained to lead cardiac arrest teams in an internal medicine study because such events were too rare (4). Pediatric residents, who are less exposed to resuscitation than internal medicine residents (5), also report a lack of clinical skills when confronted to pediatric cardiac arrest (6).

To improve exposure to these situations, experts have recommended in-hospital simulation-based trainings (7). Simulation-based trainings have been shown to be effective in teaching clinical skills, improving pediatric residents confidence (8), and improving pediatric survival rate (9).

However, according to the European Resuscitation Council, non-clinical skills, which include team communication and leadership, are as important as clinical skills for patient outcomes (10, 11). Team communication, interdisciplinary collaboration, and good leadership have shown a favorable impact on resuscitation outcome (11–15). TeamSTEPPS defines leadership as the “ability to direct and coordinate the activities of other team members, assess team performance, assign tasks, develop team skills and attitudes, motivate team members, plan and organize, and establish a positive atmosphere” (16). These abilities imply a hands-off position for the leader described as a benefit for resuscitation (17). Grant et al. defined 12 non-clinical skills in their Evaluation Score (18), which cover four concepts: physical and verbal leader's position; communication and delegating skills; ability to reassess, adapt and anticipate; and ability to ask for internal and external help.

Non-clinical skills are taught during advanced life support certification (19), but might have been less frequently applied in simulation-based trainings (6). Initiatives to improve this situation, such as leadership-oriented feedbacks after simulation-based trainings and specific leadership workshops have been shown to improve non-clinical skills (20–22). To further bolster leadership skills, other techniques have been evaluated. Some authors have evaluated the effect of blindfolding the leader, although not in a randomized controlled trial (23, 24). Our objective is to evaluate the effect of blindfolding the team leader on leadership skills, using a randomized controlled trial design.

## Method

### Population

The study is a randomized controlled trial, which took place in the Pediatric Simulation Center of the Geneva University Hospital, the second biggest pediatric tertiary university hospital of Switzerland. As no patient was enrolled, this study evaluating teaching strategies, our local institutional review board (Commission d'Ethique de la Recherche de Genève) waived the need to submit our protocol. However, each participant signed a written informed consent.

Eligible participants were all pediatric emergency fellows, residents and nurses. Exclusion criteria were 1) inability to participate in the full 4-h session, or 2) prior knowledge of the simulations scenarios. Participants were recruited among the staff of the pediatric emergency department. They were assigned to 12 resuscitation teams of four members: one emergency fellow, one emergency resident, and two pediatric registered nurses. These 12 teams were randomly allocated following simple randomization procedure (sealed envelopes with a 50% chance) to the experimental blindfold group (BG) and the control group (CG).

Participants were given a $100 gift-card.

### Intervention

**Figure 1:**
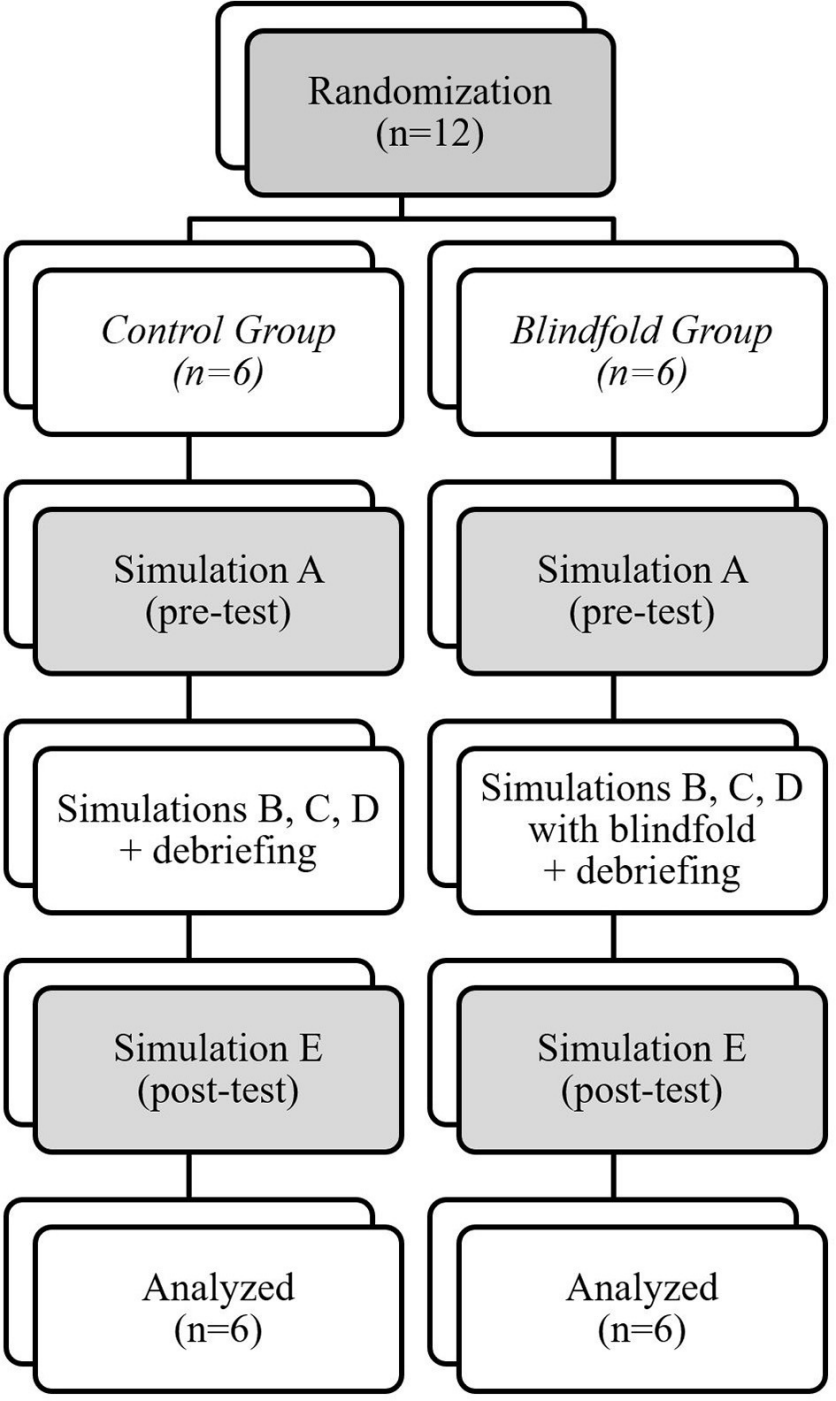
Randomization.

All participants took part in a 4-h session of five high-fidelity simulation-based scenarios, labeled A, B, C, D, and E (Table 1 and Figure 1). All the scenarios were designed by advanced pediatric simulation specialists, trained by EuSim (www.EuSim.org), and were intended to be easily managed when following the Pediatric Advanced Life Support (PALS) algorithms.

**Table 1:** Description of the simulation scenarios.

**Simulation**	**Description**	**Conditions for resolution**
A	Pulseless electrical activity secondary to severe dehydration in a 6-month old infant	CPR and two doses of epinephrine
B	Cardiac arrest on ventricular fibrillation after electrocution in a 14-month old child	CPR, defibrillation, epinephrine and second defibrillation
C	Unstable supra-ventricular tachycardia in a 12-month old child	Adenosine and synchronized defibrillation
D	Unstable Bradycardia at 65 bpm after unknown intoxication (beta-blockers) in a 10-month old child	Epinephrine and either transthoracic pacing or treating the underlying cause (information given if asked after primary assessment)
E	Asystole in a 4-month old infant	CPR and two doses of epinephrine

*CPR, cardio-pulmonary resuscitation*.

Simulations A and E were the pre-test and the post-test simulations for both groups and was not followed by a debrief (Figure 1). These scenarios were comparable to avoid additional difficulties, which could have affected the non-clinical skills. Immediately after these simulations, each team member was asked to answer two questionnaires on their perceived stress and their satisfaction of the quality of the resuscitation (Data sheets 1 and 2).

Scenarios B, C, and D were identical for both groups. The intervention was using a blindfold for the BG leader for the scenarios B, C, and D. The blindfold was a commercial sleep mask blocking out light. There was no blindfold use in the CG. Each simulation was followed by a standardized debrief lasting a maximum of 20 min, provided by 2 senior Advanced EuSim Instructors, without video feedback. The debrief focused on the team leader's non-clinical skills, in particular the team leader's spatial and verbal position, his communication and delegation skills, and his ability to reevaluate, adapt, anticipate and to ask for help. Every topics were discussed in each group after these three training simulations (Figure 1).

### Outcome

The primary outcome was the progression of the Resuscitation Team Leader Evaluation score between pre-test (simulation A) and post-test (simulation E). For the purpose of this study, we removed the items 1 and 12 from the score described by Grant et al. (18), as the leader was clearly defined from the beginning of the study and teams did not have the possibility to ask for external help. Therefore, the non-clinical skills were assessed on a scale of 0 to 30 points.

Simulations A and E video recordings were scored by three simulation-based training experts who did not take part to the simulations. The experts were blinded to the team allocations to CG or BG.

Secondary outcomes assessed by the experts were: time to CPR from cardiac arrest (measured from the circulatory arrest to CPR initiation), number of pertinent “huddles” (or reassessments), and number of complete and incomplete communication loops. We defined a communication loop as complete when three elements were present: the leader identifies a team member by name, they respond with an audible confirmation of the order, and finally verbally confirm when the task is completed. Finally, we evaluated the difference between pre- and post-test perceived stress (25) and the pre- and post-test satisfaction questionnaires.

We collected the participants' demographic data (number of years of pediatric emergency experience, number of participated sessions of pediatric high-fidelity simulation, number of years from the last PALS certification) to assess for comparability among groups.

### Sample Size

The sample size was based on a study that used the Resuscitation Team Leader Evaluation (20), where the mean non-clinical score was 15.6 ± 2.0 points (out of a maximum of 30 points). Based on an estimated 25% improvement from baseline (3.9 points) in the CG and a 50% improvement (7.8 points) in the BG, with a 90% power and an alpha of 0.05, the sample size was six teams in each group.

### Statistical Analysis

Results are presented using medians and interquartile ranges (IQR), or numbers and proportions. Differences between the two groups were assessed using Mann-Whitney *U*-tests or Fisher's exact tests, as appropriate.

All tests were two-sided, with a level of 0.05. We used SPSS version 22 for Mac (SPSS, Chicago, IL) for all statistical analyses.

## Results

### Population

From December 2016 to March 2017, we recruited 48 participants (12 pediatric emergency fellows, 12 pediatric emergency residents and 24 pediatric emergency registered nurses), who were assigned to 12 teams. There were no statistically significant differences between the BG and CG characteristics at baseline (Table 2).

**Table 2:** Demographic comparison between groups.

**Variables**	**Control group (IQR)**	**Blindfold group (IQR)**	***p-*value**
Experience of leader [yr]	2.5 (1.6;5.0)	1.5 (0.1;2.5)	0.20
Leader's number of simulations	14 (6;15)	9 (3;16)	0.68
Leader's nb of years from last PALS [yr]	3.0 (1.8;3.0)	6.0 (1.0;8.0)	0.25
Resident's experience [yr]	1.0 (0.6;2.4)	0.8 (0.4;2.1)	0.63
Resident's number of simulations	3 (2;5)	4 (3;5)	0.22
Resident's nb of years from last PALS [yr]	0.5 90.0;1.25)	1.5 (1.0;5.3)	0.06
Nurse #1's years of experience [yr]	7.0 (1.7;9.2)	10.5 (3.0;14)	0.13
Nurse #1's number of simulations	8 (3;13)	8 (4;12)	0.94
Nurse #1's number of years from last PALS [yr]	0.0 (0.0;2.8)	0.5 (0.0;2.0)	0.79
Nurse #2's years of experience [yr]	5.0 (3.5;7.3)	9.0 (4.1;11.5)	0.26
Nurse #2's number of simulations	8 (5;10)	8 (4;10)	0.81
Nurse #2's number of years from last PALS [yr]	0.0 (0.0;5.0)	0.0 (0.0;6.6)	0.70

*IQR, interquartile ranges; yr, year; PALS, Pediatric Advanced Life Support*.

### Outcomes

**Figure 2:**
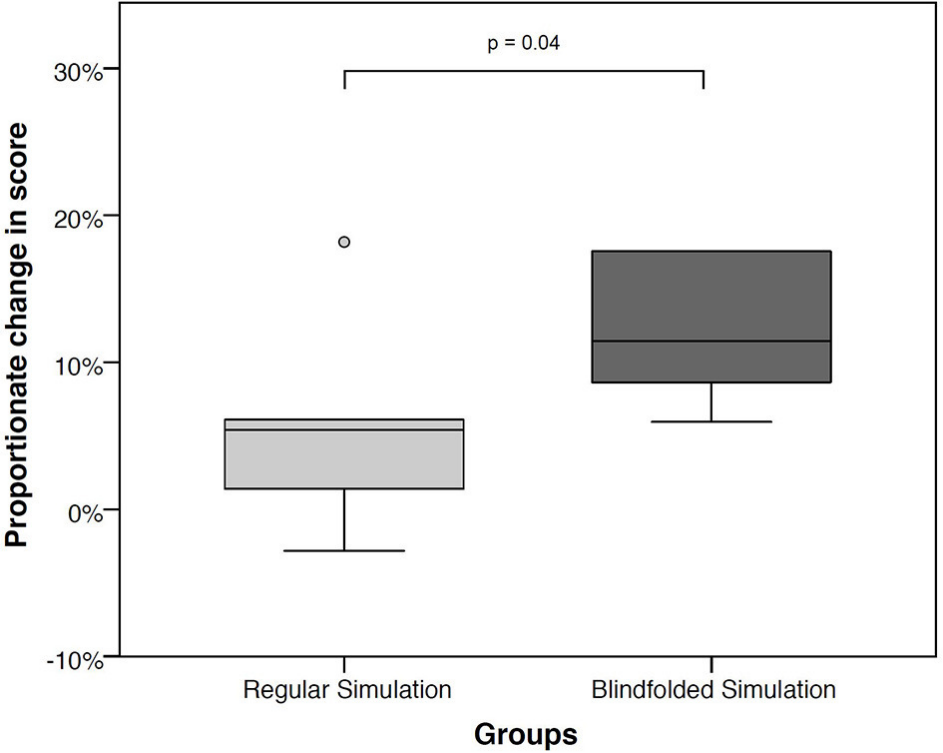
Proportional change in Calgary score from baseline, between regular and blindfolded simulation.

The Calgary score did not differ significantly between the two groups at baseline: BG 19 points (IQR 13;27) vs. CG 24 points (IQR 18;27), *p* = 0.57. The change in Calgary score was significantly greater in the BG than the CG at the end of the session: 11.4% (IQR 8.0;18.9) vs. 5.4% (IQR 0.0;8.6), *p* = 0.04 (Tables 3, 4, and Figure 2).

**Table 3:** Outcomes for blindfolded and control groups.

**Variables**	**Control group Median (IQR)**	**Blindfold groupMedian (IQR)**	***p-*value**
**BASELINE**			
Baseline Calgary score (over 30 points)	24 (18;27)	19 (13;27)	0.57
**PRIMARY OUTCOME**			
Proportion of improvement	5.4% (0.0;8.6)	11.4% (8.0;18.9)	0.04
**SECONDARY OUTCOMES**			
Change in time to cardio-pulmonary resuscitation [sec]	61 (17;151)	190 (58;267)	0.15
Change in complete communication loops [number]	+3 (−1;4)	0 (−7;5)	0.63
Change in uncomplete communication loops [number]	0 (−2;0)	−2 (−4;−1)	0.05
Change in reassessments [number]	0 (−2;3)	1 (−1;3)	0.57
**PERCEIVED STRESS AND SATISFACTION**			
Change in Leader's stress	2 (0;10)	0 (−5;4)	0.38
Change in Intern's stress	−1 (−10;3)	−7 (−13;−5)	0.17
Change in Nurses' stress	−15 (−16;−11)	−12 (−30; −10)	0.34
Change in Leader's satisfaction	5 (0;17)	3 (1;22)	0.98
Change in Resident's satisfaction	9 (3;16)	14 (6;24)	0.70
Change in Nurses' satisfaction	24 (14;25)	23 (7;38)	0.21

*IQR, interquartile ranges. All changes are calculated between Simulation A (pre-test) and Simulation E (post-test)*.

**Table 4:** Changes in the calgary score for blindfolded and control groups.

**Calgary score items**	**Control group Median (IQR)**	**Blindfolded group Median (IQR)**	*P-*value^*^
Delegates roles and responsibilities to team members	−0.6 (−1.0;0.0)	−0.5 (−0.7; −0.1)	0.62
Maintains control of leading the resuscitation	0.0 (−0.1;3.3)	0.5 (0.2;1.3)	0.04
Uses effective closed loop communication	0.0 (0.0;0.4)	0.3 (0.2;0.8)	0.13
Manages team resources and distributes workload appropriately	0.0 (−0.1;1.1)	0.3 (−0.1;1.4)	0.66
Verbalizes thoughts and summarizes progress periodically for benefit of team	0.3 (0.0;1.2)	0.2 (0.0;1.0)	0.93
Asks for and acknowledges input from team members	0.2 (−0.4;0.8)	0.2 (−0.1;0.4)	0.99
Reassesses and reevaluates situation frequently	0.2 (−0.2;0.8)	0.5 (0.3;1.0)	0.37
Avoids fixation errors	0.3 (0.2;0.4)	1.0 (−0.2;1.7)	0.29
Refrains if possible from active participation	0.0 (−0.4;0.4)	0.3 (−0.1;1.1)	0.25
Shows anticipation of future events by asking for preparation of equipment or medication not yet needed	0.0 (−0.1;0.5)	0.0 (−0.1;0.7)	0.99

*IQR, interquartile ranges*.

*All changes are calculated between Simulation A (pre-test) and Simulation E (post-test)*.

* *P-values are not corrected for multiple comparisons*.

Although there were no differences in the number of complete communication loops, there was a significant decrease in the number of incomplete communication loops in the BG (*p* = 0.05). There were no statistically significant differences in time to CPR between the two groups (*p* = 0.15) (Table 3).

There was no significant change in the leaders' and teams' stress nor in the self-reported satisfaction score (*p* = 0.98; Table 3).

## Discussion

The improvement in leadership skills after the three blindfolded simulations corroborates with the findings from previous non-randomized observations (23, 24). This might be explained by an overall enhancement of teamwork skills, in particular for leadership. Blindfolding the leader might help improve leadership skills by helping keep a distance, instead of focusing on details or taking part in the actions, such as performing chest compressions. Although sometimes unavoidable due to lack of resources, taking part in the actions as a leader can lead to delays in responsiveness to changes to new information such as a change in the patient's state. Furthermore, emergent situations are often noisy with lots of distractors. Wearing a blindfold might help the leader avoid distractions, and can help him focus on the “numbers” or on following the right algorithms. Future studies could help to specify why blindfolded simulations seem to improve leadership skills. For example, it might be interesting to use eye-tracking techniques to analyze the leader's gaze (26). This could allow us to study the time the leader spends focusing on the monitor screen or micromanaging other team members' tasks.

Having a blindfolded leader also requires other team members to improve their communication skills overall. First, all the important data need to be verbalized to keep the leader informed about the current events. Basically, this is calling out a selection of pertinent events or data. Second, team members need to take turns when communicating, because the leader will not be able to follow if they all speak at once. Finally, team members need to acknowledge requests for action, since the leader cannot see if his instructions have been heard. All of these communication tools are important for collaborative teamwork (16). In our study, we found a significant improvement for incomplete communication loops.

We also studied the effect of blindfold on stress. Stress is known to be a major cause of cognitive impairment (27), and one could hypothesize that having to lead a situation, and bear responsibility for the outcome, without being able to see may engender additional stress. However, in light of the discussion above about blindfolds helping to be more focused on the CPR algorithms, and less distracted by the surrounding noise and activity, the leader's perception of stress may in fact *benefit* from wearing a blindfold. For the other members of the team, stress may be affected by the trust placed in a blindfolded leader's instructions. In our sample, neither the leader's or other team members' stress was increased by the use of a blindfold.

Some limitations for our study must be recognized. First of all, the leadership progression isn't as high as expected. Even if our results are statistically significant, one might question its clinical significance. Also, the two groups had different baseline scores. Nevertheless, we believe that this should not have impacted our results, as the effect of the intervention was calculated as a change from baseline. The debriefing sessions might also have been influenced by the allocation to one arm or the other, because the simulation instructor was not blinded to the allocation as he had to observe the simulations. To address this potential bias, our debriefings were strictly scripted and monitored by the primary investigator who was present for all simulations and debriefings. Another limitation is the variability in the resuscitation team, as the junior physician and the two nurses had heterogeneous levels of experience and training. Other studies have standardized the team, using the same team for all simulations (20). However, heterogeneous teams are more pragmatic and improve the generalizability of our results. Furthermore, as debriefings were aimed at leadership skills, results might be influenced by this consecutive effect. However, this effect should have been minimized by the fact that both groups had similar standardized debriefings addressing leadership issues. Additionally, a more homogeneous team would have increased the statistical power, as it would have reduced the “noise” in the statistical analysis. Our study was also limited by the fact that blindfolded simulation does not represent reality. However, blindfolded simulation seems to be efficient even if it not realistic, as is the case of other teaching technics, such as serious games. Moreover, our sample size was calculated based on an anticipated effect of the intervention that was higher than the one observed. This might explain why most of our secondary outcomes are not statistically significant. We must also acknowledge that the satisfaction was measured with a non-validated tool. Finally, post-training assessment took place just after training; it would have been interesting to propose a follow-up assessment at 3 or 6 months.

## Conclusion

Blindfold might be an efficient method for leadership training during pediatric resuscitation simulated scenarios. Future studies should further assess the effect of blindfolded training on communication skills and on clinical outcomes after pediatric resuscitation.

## Author Contributions

MB and OK initiated the study. MB and OK have full access to all of the data in the study and take responsibility for the integrity of the data and the accuracy of the data analysis. All authors contributed to design the study, organization of data acquisition and to interpretation of the results. The first draft of the manuscript was written by MB and OK. All authors contributed to amend it and approved the final version.

### Conflict of Interest Statement

The authors declare that the research was conducted in the absence of any commercial or financial relationships that could be construed as a potential conflict of interest.
