# Supplementary material for: Effects of Blindfold on Leadership in Pediatric Resuscitation Simulation: A Randomized Trial
Source: Front Pediatr. 2019 Feb 14;7:10. doi: 10.3389/fped.2019.00010 (PMC6383074; doi:10.3389/fped.2019.00010)
Supplement: Supplementary file 1 [file Data_Sheet_1.PDF]

Votre perception de stress

(questionnaire basé sur l'étude Perceived Stress in a probability sample, Cohen & Williamson, 1988)

Vos initiales :

Questionnaire après : □ scénario A (pré-test) □ scénario E (post-test)

Merci de remplir ce questionnaire pour évaluer votre perception de stress pendant la simulation de réanimation à laquelle vous venez de participer. Pour cela, mettez une croix dans la bulle correspondant au niveau de véracité de la proposition selon votre ressenti.

Au cours de cette simulation de réanimation…

	pas du tout										énormément
vous êtes-vous senti(e) incapable de contrôler les choses importantes ?	○	○	○	○	○	○	○	○	○	○	○
vous êtes-vous senti(e) nerveux(se) et stressé(e) ?	○	○	○	○	○	○	○	○	○	○	○
vous êtes-vous senti(e) confiant(e) dans votre capacité à prendre en main la situation ?	○	○	○	○	○	○	○	○	○	○	○
avez-vous senti que les choses allaient comme vous le vouliez ?	○	○	○	○	○	○	○	○	○	○	○
avez-vous trouvé que vous ne pouviez pas assumer toutes les choses que vous devriez faire ?	○	○	○	○	○	○	○	○	○	○	○
vous êtes-vous senti(e) en colère à cause de choses qui étaient en dehors de votre contrôle ?	○	○	○	○	○	○	○	○	○	○	○
avez-vous senti que les difficultés s'accumulaient à un tel point que vous ne pourriez les surmonter ?	○	○	○	○	○	○	○	○	○	○	○

Stress questionnaire
English translation

Please complete this questionnaire to assess your perception of stress during the simulation you just participated in.

During this resuscitation simulation,

	Not at all		Very much
Did you feel unable to control the important things?			
Did you feel nervous and stressed?			
Did you feel confident in your ability to take charge of the situation?			
Did you feel things were going the way you wanted?			
Did you find that you could undertake all the things you had do?			
Did you feel angry at things that were outside your control?			
Did you feel that the difficulties accumulated to a point that you could not overcome?			
