# Supplementary material for: Effects of Blindfold on Leadership in Pediatric Resuscitation Simulation: A Randomized Trial
Source: Front Pediatr. 2019 Feb 14;7:10. doi: 10.3389/fped.2019.00010 (PMC6383074; doi:10.3389/fped.2019.00010)
Supplement: Supplementary file 2 [file Data_Sheet_2.PDF]

Votre ressenti et votre niveau satisfaction sur la simulation

Vos initiales :

Questionnaire après : □ scénario A (pré-test) □ scénario E (post-test)

Merci de remplir ce questionnaire pour évaluer votre ressenti et votre niveau de satisfaction en regard de la simulation de réanimation à laquelle vous venez de participer. Pour cela, mettez une croix dans la bulle correspondant au niveau de véracité de la proposition selon votre ressenti.

Pendant cette simulation…

	pas du tout										énormément
A quel point vous êtes-vous senti à l'aise dans la collaboration avec d'autres corps de métier (médecins, infirmiers) ?	○	○	○	○	○	○	○	○	○	○	○
A quel point <u>votre performance individuelle</u> est-elle adéquate ?	○	○	○	○	○	○	○	○	○	○	○
A quel point <u>votre fonctionnement</u> dans l'équipe est-il adéquat ?	○	○	○	○	○	○	○	○	○	○	○
A quel point la collaboration de l'équipe pluridisciplinaire (médecins, infirmiers) était-elle satisfaisante ?	○	○	○	○	○	○	○	○	○	○	○
A quel point la répartition des tâches au sein de votre équipe était équilibrée ?	○	○	○	○	○	○	○	○	○	○	○
A quel point la communication au sein de votre équipe était suffisante ?	○	○	○	○	○	○	○	○	○	○	○
A quel point l'équipe s'est montrée efficace à trouver des solutions aux problèmes cliniques rencontrées ?	○	○	○	○	○	○	○	○	○	○	○
A quel point vous êtes globalement satisfait de l'équipe ?	○	○	○	○	○	○	○	○	○	○	○

Satisfaction questionnaire
English translation

Please complete this questionnaire to assess your satisfaction during the simulation you just participated in.

During this resuscitation simulation,

	Not at all		Very much
How comfortable did you feel in working with other disciplines (doctors, nurses)?			
Was your own performance adequate?			
Did your team work appropriately?			
How was the collaboration of the multidisciplinary team (doctors, nurses)?			
How balanced was your team's workload?			
How well did your team communicate?			
How effective was the team in finding the solutions to the clinical challenges you encountered?			
